# Severe Acute Kidney Injury Secondary to Immunoglobulin Infusion in Life-Threatening Guillain Barre Syndrome

**DOI:** 10.30476/BEAT.2021.85702.1103

**Published:** 2022-01

**Authors:** José David Orquera, María Marta Pernasetti, Patricia Ojeda, Griselda Agüero, Daniel Agustín Godoy

**Affiliations:** 1 *Neurointensive Care Unit, Sanatorio Pasteur, Catamarca, Argentina*; 2 *Department of Nephrology, Sanatorio Pasteur, Catamarca, Argentina*; 3 *Department of Neurology, Sanatorio Pasteur, Catamarca, Argentina*

**Keywords:** Guillain Barre Syndrome, Acute Flaccid Paralysis, Immunoglobulin, Immunotherapy, Acute kidney injury (AKI).

## Abstract

Immunoglobulin infusion (IVIG) is one of the first line therapy in Guillain Barre Syndrome (GBS). Several medical complications are associated with GBS (pneumonia, sepsis, deep vein thrombosis, dysautonomy). Acute kidney injury (AKI) is an uncommon complication during IVIG infusion. Several risk factors were associated with AKI during IVIG. These are an older age, previous renal disease, concomitant use of nephrotoxic agents, diabetes mellitus, hypovolemia, sepsis or using of IVIG that contained in its preparation sucrose or mannitol as stabilizers to avoid precipitation and aggregation. Infusion rate and total dose play a determinant role. The most important pathophysiological mechanism of AKI are the osmotic stress applied to the epithelium of proximal tubules and glomeruli. The osmotic overload is principally generated by IVIG stabilizers (sucrose). In general, AKI is reversible but approximately 30% hemodialysis is necessary. It is essential to respect doses, infusion rates and closely monitoring renal function parameters during IVIG infusion.

## Introduction

Guillain-Barre syndrome (GBS) was first described in 1859, however, it was recognized as such in 1916 [1, 2]. It is an inflammatory and autoimmune disease of the peripheral nerves being the most common cause of acute or subacute flaccid paralysis [1, 2]. Its annual incidence is 1-2 per 100,000 inhabitants. There are different clinical forms which characterized by accelerated progression that reach within 2 weeks of the onset of symptoms [3]. Approximately one fifth of individuals will develop respiratory failure with the need for artificial airway and mechanical ventilation [1, 2]. Additionally, the autonomic nervous system compromise can lead to different types of arrhythmias and hemodynamic instability. Both situations can be life-threatening in the acute phase with an estimated incidence close to 10% [1, 2]. In general, the clinical course of GBS is monophasic with an initial phase of progression followed by a plateau of variable duration from days to months before entering the recovery phase [1-3]. The basis of the therapy is immunotherapy either by intravenous immunoglobulin (IVIG) infusion or plasmapheresis [1, 2]. Individuals are subjecting to multiple and varied complications during the course of the disease. To those already mentioned are added those of prolonged intensive care (pneumonia, sepsis, malnutrition, pressure ulcers, deep vein thrombosis, electrolytic disturbances) or of the employed therapy [1, 2]. In this context, acute kidney injury secondary to IVIG infusion is an uncommon possibility and reason for reporting the present case.

## Case Presentation

A 48-year-old woman with bipolar disorder undergoing treatment with levomepromazine and clonazepam presented to the emergency department due to progressive, bilateral and symmetrical weakness of both lower limbs leading to falls, and accompanied by paresthesias on both feet. She reported a history of aqueous diarrhea of an evolution week. Severe hypokalemia (K + 1.8 mEq / L) was detected with electrocardiographic alterations (ST segment depression, flattening of T waves and U wave presence). She was admitted to the Neurointensive Care Unit. 

Urinary K+ was within the normal range (23 mEq / L) in a random sample. On the other hand, [complete abbreviation] (VBG) were obtained after verifying the adequate placement of the venous line. pH: 7. 37, paCO2: 46mmHg, pvO2: 42mmHg; HCO3- 22.5 mEq / L Hypokalemia was attributed to gastrointestinal symptoms. Hyperaldosteronism, Cushing’s syndrome, drugs (diuretics, B2 agonists), hypomagnesemia, etc. were ruled out. 

Her vital signs were normal. Blood pressure was 125/75 mmHg, rectal temperature 36.3 ° C, and her respiratory and cardiac rates were18 and 88, respectively. Neurological examination showed a lucid and oriented patient with intact extraocular movements and significant paraparesis. The onset of weakness was distal with proximal progression; deep tendon reflexes were absent. No sensory level was detected. Peri-oral and lower extremity paresthesias extending to the lower third of both legs were present. Examination of cranial nerves showed no involvement. Cough and swallow reflexes were preserved. 

Ventilation was normal with an SaO2 of 95% at ambient air. Chest x-ray was normal. Potassium replacement was started but the clinical picture remained unchanged. The rest of the laboratory values were normal except for the presence of leukocytosis (14,800 / mm3), C-reactive protein increase (61 mg / L) and mild metabolic acidosis. Renal function was intact with urea 30 mg/dL and creatinine 1.1 mg/dL. 

Supplemental potassium was initiated through intravenous infusion with loading and maintenance doses, normalizing to a value of 4.1 mEq / L, after which the electrocardiogram (EKG) alterations were normalized, but not the neuromuscular compromise. 

There was weakness progression to both arms at 20 hours after admission. Diffuse areflexia was maintained. Tactile and thermoalgesic sensitivity was preserved throughout the whole body. Paresthesias in hands and around the mouth were present. Dysphagia were present with a weak cough and swallow reflexes. The patient was unable to count until 10. Alveolar hypoventilation and hypoxemia were present in blood gases (paC02 57 mmHg; paO2 58 mmHg at 40% of inspired oxygen fraction). Orotracheal intubation and mechanical ventilation was initiated. Due to GBS clinical suspicion, a lumbar puncture was performed which showed albumin-cytological dissociation (1 cell / mm3; 96 mg / dL proteins). Electromyogram (EMG) of upper and lower limbs was compatible with acute inflammatory demyelinating polyradiculoneuropathy characterized by prolonged distal motor latencies in both tibial and peroneal muscles together with slowing and conduction block in both upper and lower extremities The explored latencies of F waves were prolonged. The sural response was normal.

After the GBS diagnosis was confirmed, a human intravenous Igb (manufactured by Cordoba National University, Cordoba, Argentina) course was instituted at a rate of 400 mg/kg/day for 5 days. The patient was mechanically ventilated under midazolam sedation and fentanyl analgesia. Autonomic dysfunction leads to hemodynamic instability requiring norepinephrine support and adenosine for the episodes control of supra-ventricular arrhythmias.

On the fifth day of admission, there was a significant drop in urine output but remained at 40 ml / hour. Urea and creatinine were increased to 147 and 4.8 mg / dL respectively, and metabolic acidosis (pH 7.27; bicarbonate 14 mEq / L) and hyperchloremia (Cl^-^ 123 mEq/L) and hypernatremia (Na + 160 mEq / L) were present. 

Anion gap was calculated: (160+3.5)-(123+14)=26.5 mEq/L and was interpreted as hyperchloremic metabolic acidosis, compatible with acute kidney injury (AKI). In addition, causes such as lactic acidosis (0.9 mmol / L), diabetic ketoacidosis (normal blood glucose levels, absence of ketone bodies in blood and urine); drugs (paracetamol, acetazolamide), absence of urethral diversion; and tubular acidosis were ruled out. 

Acute kidney injury was diagnosed following conventional criteria [4, 5]. Volume status was preserved (CVP 10 mmHg with conserved diameter of inferior vena cava in ultrasound). No nephrotoxins have been administered. Only crystalloids fluids (normal saline and 5% dextrose) were employed.

Urine sediment evidenced the presence of granular and pigmented casts with epithelial cells. Absence of hyaline and hematic casts, bacteria or fungi. 

**Fig. 1:**
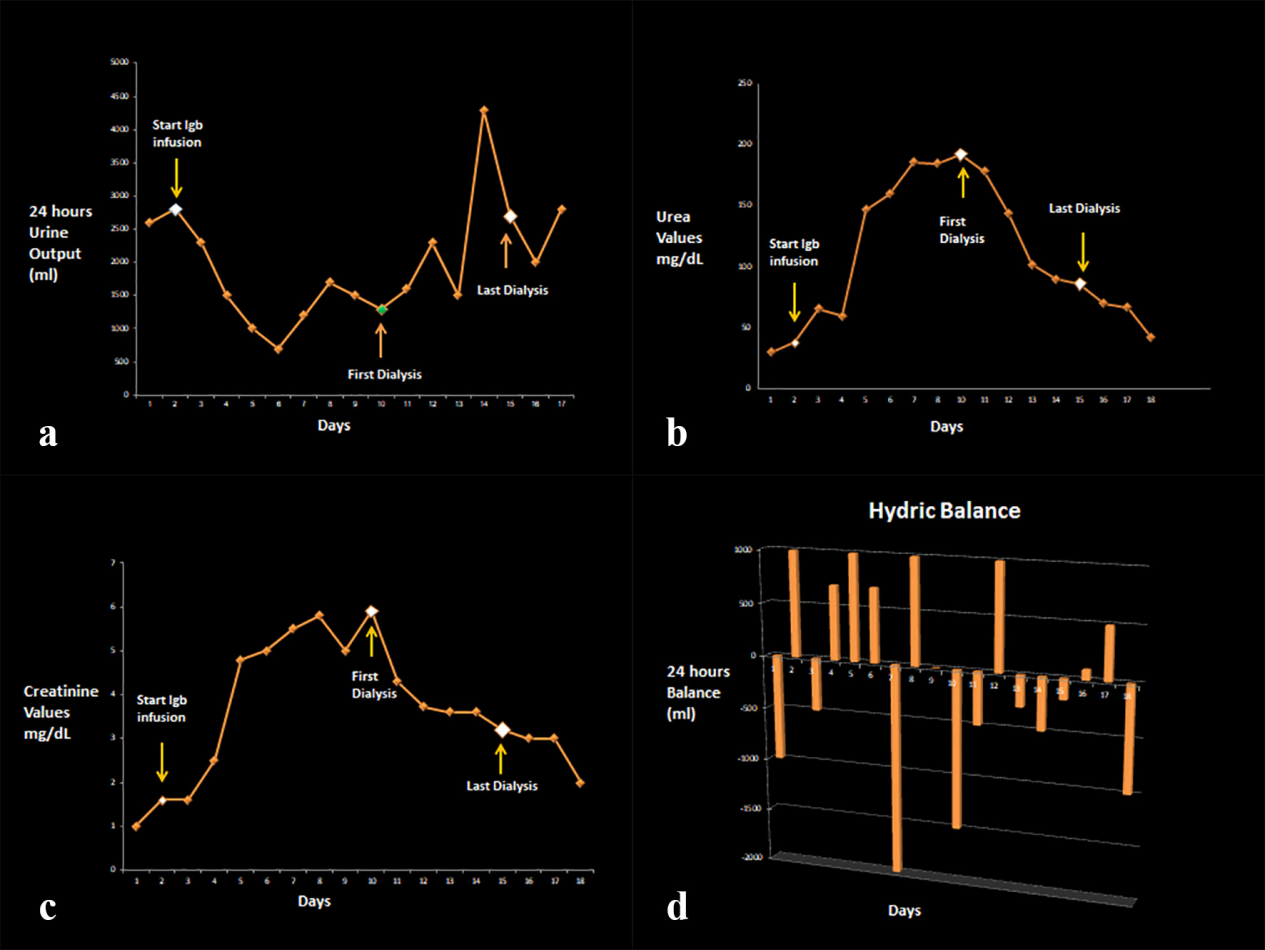
**(A)** 24 hour-diuresis, **(B)** urea, **(C)** creatinine values and **(D)** water balance evolutionary profile

Normal renal size and morphology was present in renal ultrasound with preservation of the cortical/medullary ratio. The Nephrology service suggested to reduce the IVIG infusion to half the rate; the infusion was completed at the 8th day of admission. Urine output was maintained and lower doses of vasopressors were needed. Progressive renal dysfunction was noted (Figure 1). Mechanical ventilatory support continued and percutaneous tracheostomy was performed.

At 10^th^ day hemodialysis was started due to the progressive deterioration of renal function. Progressive correction of metabolic acidosis and hypernatremia accompanied with gradual decrease in creatinine and urea values were achieved. The patient remained hemodynamically stable and vasopressors were weaned off at day 12. At this point, isolated febrile episodes and a new pulmonary infiltrate were evident. Positive bronchial lavage to Klebsiella pneumoniae was detected. Targeted antibiotic therapy was started. 

At the day 15, the patient was lucid, oriented and calm. Her vital signs were normal. She was weaned off mechanical ventilation. She had a urine output greater than 50 ml/hour with progressive decrease in urea and creatinine values. Its acid base status and electrolytes were normal. Hemodialysis was stopped.

At the day 17, she was breathing unassisted with normal arterial gases and pneumonia in resolution. Vigorous cough and swallowing reflexes was present with an effective handling of bronchial secretions. Distal motility and legs and arms strength were slowly improved. Tracheostomy cannula removal was performed. She was discharged to the general ward 20 days after admission. At the day 30, she was transferred to a comprehensive neuromuscular rehabilitation center.

## Discussion

GBS is an acute and severe entity with a monophasic but prolonged course, and potential for multiple complications [1, 2]. The origin of them is varied; while some are characteristic of neuromuscular (respiratory failure) or autonomous nervous system involvement (dysautonomy), others derive from intensive care (deep vein thrombosis, pneumonia, sepsis) or from the therapeutic measures employed (immunotherapy) [1, 2]. Acute kidney injury (AKI) is one of them [6-9]. Its etiology is multifactorial.

In general, AKI is a known but uncommon complication in the context of GBS [1, 2]. It has been described when GBS is associated with certain infections (leptospirosis, hepatitis, mycoplasma pneumoniae); autoimmune diseases (thrombocytopenic purpura, systemic lupus erythematosus, hemolytic anemia); Igb A nephropathy or IVIG infusion [6, 7].

The exact incidence of AKI associated with IVIG is not known, being estimated at less than 1% [6-9]. The diagnosis is one of exclusion and is associated more with the total dose than the infusion rate [6, 7]. The risk factors described for the development of AKI by IVIG are age> 65 years, previous renal involvement, concomitant use of nephrotoxic agents, diabetes, hypovolemia, sepsis or the use of IVIG that contained in its preparation sucrose or mannitol as stabilizers [6-9].

In this report, the patient is a young adult who presented with bulbar, respiratory and autonomic involvement, triggered by campylobacter jejuni infection. She had no history of diabetes or kidney disease and renal function was normal at admission. Nephrotoxic agents were not employed at any time. Hypovolemia, sepsis or severe and sustained arterial hypotension prior to the development of AKI were absent. The only causative factor detected was the infusion of vasopressors (norepinephrine) but at low doses (maximum employed 0.8 µ / kg / min) and IVIG at the doses and infusion rates (1 ml / min) typically recommended [1, 2].

A possibly pre-renal component was present due to secondary dehydration to vomiting and diarrhea. Notwithstanding this, the central line was made upon admission. CVP was 2 cmH2O and the ultrasound of the inferior vena cava showed inspiratory collapse. These parameters were normalized after initial resuscitation with normal saline (2.5 liters). 

Rhabdomyolysis was ruled out from admission due to the lack of clinical history (trauma, drugs, intense physical exercise, heat stroke, etc); absence of detectable myoglobin in blood and negative myoglobinuria in addition to the aforementioned findings in urinary sediment and normal CPK and aldolase levels (37 mcg / L and 2.1 U / L respectively) 

AKI usually develops within the initial 10 days of the start of IVIG, and in general is reversible; nevertheless in about 30% temporary hemodialysis may be required [6, 7]. Renal function tends to normalize within 6 weeks after the onset of AKI [6, 7].

Regarding its pathophysiology, multiple hypotheses have been developed such as glomerular precipitation of immune complexes, renal ischemia and tubular damage secondary to the osmotic stress to which the epithelium is subjected due to preservatives such as sucrose, or mannitol [6-9].

Pathological studies reveal vacuolization and swelling of the proximal tubular epithelium and to a lesser extent of the glomeruli which is similar to the damage caused by mannitol [6-9].

The IVIG preparation used in the reported case were included sorbitol and glycine in order to avoid the aggregation of IVIG and its adverse effects. Although sorbitol has not been specifically reported as a cause of AKI, it is a polyhydric sugar alcohol obtained by reduction of glucose catalytic hydrogenation. Its association has been described with adverse effects (not AKI) in individuals with hereditary fructose intolerance [7].

Biopsy was not performed and it was not considered necessary to perform this invasive procedure due to the very good clinical response. 

## Conclusion

In conclusion, the use of IVIG is emerging as a first line therapy in GBS due to availability and ease of access. For this reason, knowledge of its adverse effects is imperative especially in risk populations. During infusion, intravascular volume depletion and the use of nephrotoxic agents should be avoided, as well as, respecting the infusion rates recommended by each manufacturer. Renal function should be monitored closely during infusion. If possible, especially when the risk is very high, sucrose-free formulations should be used. It has been reported that IVIG with maltose is well tolerated as a preservative and rarely compromises renal function mainly due to the fact that maltose is metabolized by renal cells which is not the case with sucrose [6, 7]. Finally, we recommend the early using of hemodialysis when necessary.

## Declarations

### Ethics approval and consent to participate:

The manuscript received the approval of the ethics committee of the institution following the guidelines of the Helsinki declaration. The patient and his family members gave their signed consent for the publication.

### Consent for publication:

All the authors gave their consent for the publication.

### Conflict of interests:

There is no conflict of interest.

### Funding:

None.

### Authors' contributions:

All authors participated in the same way in the design, bibliographic search and writing of the manuscript.

### Acknowledgements

Special thanks to the nursing staff of the Neurological Intensive Care Unit of the Pasteur Sanatorium.
